# Microbial Sensing by the Intestinal Epithelium in the Pathogenesis of Inflammatory Bowel Disease

**DOI:** 10.4061/2010/671258

**Published:** 2010-06-29

**Authors:** Michael Scharl, Gerhard Rogler

**Affiliations:** Division of Gastroenterology and Hepatology, Department of Internal Medicine, University Hospital Zurich, Rämistrasse 100, CH-8091 Zurich, Switzerland

## Abstract

Recent years have raised evidence that the intestinal microbiota plays a crucial role in the pathogenesis of chronic inflammatory bowels diseases. This evidence comes from several observations. First, animals raised under germ-free conditions do not develop intestinal inflammation in several different model systems. Second, antibiotics are able to modulate the course of experimental colitis. Third, genetic polymorphisms in a variety of genes of the innate immune system have been associated with chronic intestinal inflammatory diseases. Dysfunction of these molecules results in an inappropriate response to bacterial and antigenic stimulation of the innate immune system in the gastrointestinal tract. Variants of pattern recognition receptors such as NOD2 or TLRs by which commensal and pathogenic bacteria can be detected have been shown to be involved in the pathogenesis of IBD. But not only pathways of microbial detection but also intracellular ways of bacterial processing such as autophagosome function are associated with the risk to develop Crohn's disease. Thus, the “environment concept” and the “genetic concept” of inflammatory bowel disease pathophysiology are converging via the intestinal microbiota and the recognition mechanisms for an invasion of members of the microbiota into the mucosa.

## 1. Chronic Inflammatory Bowel Diseases

**Figure 1:**
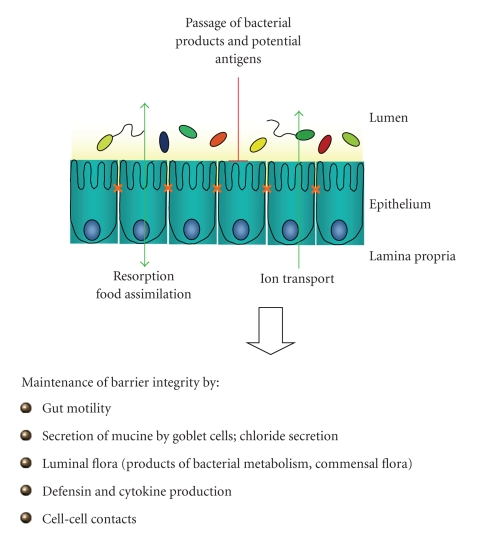
The intestinal epithelial barrier. The human gastrointestinal tract contains myriads of microorganisms. From oral to anal the number of bacteria is increasing tremendously. Especially the colon and the colonic epithelial cells are challenged by a heavily and continuous exposure to bacteria and antigens. The healthy epithelium represents a highly selective barrier that separates the body, especially the cells of the intestinal immune system, from the gut content. Therefore, it inhibits the passage of bacterial products and potential antigens and regulates the nutrient uptake as well as the resorption and secretion of ions and water. The integrity of the intestinal epithelium is maintained by a tightly controlled orchestra of regulatory mechanisms, such as the secretion of mucus, the production of defensins and cytokines, or intercellular connections.

Two major forms of chronic mucosal inflammation can be discriminated. In Crohn's disease (CD) the whole gastrointestinal tract may be involved. However, the most frequent site of inflammation is the terminal ileum, the last part of the small bowel and the adjacent caecum. In CD the inflammation affects all layers of the gut wall and frequently an alteration of the adipose tissue covering the colon or small bowel at the serosal side is found. Normal and involved areas of the mucosa can be found along the gut (so called skip lesions). This is in contrast to ulcerative colitis (UC). In UC there is a continuous inflammation only of the mucosa that always starts at the rectum. The extent of the disease may vary and sometimes only the rectum or the sigma is involved. In about 1/3 of the patients the whole colon will be inflamed (pancolitis). However, in contrast to CD the small intestine never is affected (the only exception is a so-called back-wash ileitis in severe cases of UC in which some inflammation extends to the last centimeters of the ileum). So from morphological aspects both diseases can clearly be discriminated (which, however, is not always the case in clinical practice). In addition, it has been demonstrated that there are clear differences with respect to pathophysiological mechanisms. In CD a strong genetic susceptibility can be found. A recent study again has shown the impact of genetic factors on the pathogenesis of CD by demonstrating a concordance in 63.6% among monozygotic twins, however, only 3.6% among dizygotic twins [1]. This concordance of monozygotic twins is much lower (around 6%) in UC indicating that a genetic susceptibility plays a minor role in this disease (Figure 1).

Among the environmental factors, diet and host microbiota seem to play the most important roles for the pathogenesis of the diseases. The diet affects the composition of the intestinal flora, which then may influence the disease course [2]. Some studies showed that a high uptake of carbohydrates might be associated with an increased risk for CD as well as UC [3–5]. However, results have not been unequivocally confirmed. Of notice, very high sugar uptake leads to insulin resistance finally resulting in a chronic inflammatory state [6, 7]. Shoda et al. found that the dietary changes in a Japanese population between 1966 and 1985 were associated with a strongly increasing risk for CD. In this population, elevated uptake of total fat, animal fat, and n-6 polyunsaturated fatty acids was paralleled by decreased intake of n-3 polyunsaturated fatty acids [8], which have been shown to exert anti-inflammatory effects [9]. This finding could be confirmed for UC by a prospective study in a European population [10]. Dietary components are also able to affect the intestinal microbiota, which shows a different composition in IBD patients as compared to healthy people [11].

In IBD patients not only the quantity of commensal bacteria in the intestine is reduced (about 10-fold lower compared to control patients), but also the quality and diversity of the commensal composition are altered [11–13]. Especially the number of the major classes of commensals, *Firmicutes* and *Bacteroidetes*, is reduced [11, 12]. On the other hand, the number of mucosal adherent bacteria, such as invasive *E. coli*, or Proteobacteria, such as *Enterobacteriaceae*, is increased, resulting in a so-called state of “dysbiosis” [11, 14, 15]. These pathogenic bacteria may then enhance an inflammatory response of the host intestine and hereby aggravate the intestinal inflammation. 

## 2. The Role of the Microbiota in Animal Models of Chronic Intestinal Inflammation

During the last few years significant advance has been achieved in the understanding of the pathogenesis of inflammatory bowel diseases (IBDs). It became evident that bacteria play an essential role for the initial trigger of the chronic inflammation in Crohn's disease (CD) and ulcerative colitis (UC). Sartor et al. demonstrated that certain bacterial strains such as bacteroides can induce or aggravate colonic inflammation in models such as HLA-B27 rats or IL-10 knock-out mice [16, 17]. Further it could be demonstrated in a number of different mouse models of colitis that these animals were prevented from colitis by raising them under germ-free conditions [18, 19]. In several models, monoassociation with just one bacteria was sufficient to be again able to induce colitis [20]. The cecal bacterial load was clearly correlated with the severity of disease in those animal models [21]. However, in different mouse strains different bacteria proved to be most effective in inducing colitis making it unlikely that one specific microbial pathogen would be the inducing factor of CD or UC. The concept was developed that in IBD the physiologic intestinal flora is no longer tolerated [22, 23]. 

## 3. Susceptibility Genes for IBD and the Role of the Microbiota

The insights obtained during genome wide association studies (GWASs) elucidating involved risk genes for IBD have shed new light on the interaction of bacteria with the mucosal immune system and the pathways by which the intestinal microbiota may contribute to chronic mucosal inflammation. 

The intestinal mucosa has long been seen as an organ that has mainly the function of nutrient digestion and resorption. However, the mucosa is exposed to a myriad of microbial antigens, uncountable potential pathogens, and even more nonpathogenic bacterial molecules. Due to its enormous surface area the barrier function of the intestinal mucosa may be as important as its function in nutrient absorption. It is obvious that there need to be effective defense mechanisms when the barrier becomes locally leaky. Controlled local inflammation after bacterial recognition may be regarded as crucial component of the mucosal defense system [24]. Mechanisms initiating or limiting inflammation need to be tightly regulated as they themselves might alter the mechanical barrier function [24]. On an intracellular level, pro- and anti-inflammatory signal transducers, regulatory proteins and immune effector genes represent a well-organized orchestra of agonists and antagonists. The interplay between each of the participating components needs to be exactly regulated. Functional deficiency of only one of the respective molecules may have tremendous consequences for the entire organism. During the last years evidence was found that specific single nucleotide polymorphisms (SNPs) within several genes, which may cause dysfunction of their respective protein products, are associated with the risk to develop IBD. 

Since 2001, GWAS revealed more than 30 genes that are associated with IBD [25]. Among the identified targets are genes that play an important role for immunological cell-cell interaction and signalling, such as tumour necrosis factor (TNF) [26], TNF-receptor 1 (TNFR1) [27], the interleukin-23 receptor (IL23R) [28], or interleukin-12p40 (IL12B) [29, 30]. Perhaps even more important, there are genes that are involved in the immune response to bacteria, such as the nucleotide oligomerization domain 2 (NOD2) [30, 31], the toll-like receptor 4 (TLR4) [32, 33], as well as the autophagy genes autophagy-related like 1 (ATG16L1) and immunity-related GTPase family M (IRGM) [28, 34, 35]. In addition, regulatory genes, such as the protein tyrosine phosphatase N2 (PTPN2) [29, 36] and the peroxisome proliferation-activated receptor gamma (PPAR*γ*) [37] as well as genes that are involved in cell homeostasis, such as the membrane transporters multidrug resistance gene 1 (MDR1) [38, 39] and the organic cation transporter 1+2 (OCTN1+2) [40, 41] have been found to be associated with the risk of chronic mucosal inflammation. 

The functional consequences of the respective SNPs have only been investigated to a limited extent. It is likely of course that these genetic variants alter functional properties of a specific protein resulting in a disturbed function and, finally, in an inadequate immune reaction.

## 4. Pattern Recognition Receptors—to TOLLerate or NOD

As discussed, bacteria and bacterial components play a crucial role for the onset and perpetuation of chronic intestinal inflammation. Thus, the appropriate response to bacterial stimuli plays a key role for the maintenance of intestinal homeostasis. Two groups of pattern recognition receptors (PRRs), the Toll-like receptors (TLRs), and the nucleotide oligomerization domains (NODs) have been demonstrated to be essentially involved in bacterial recognition, induction of antimicrobial factors, activation and modulation of innate as well as adaptive immune responses, and in the maintenance of intestinal epithelial barrier function. 

Though both of the PRR subgroups are ubiquitously expressed within the gastrointestinal tract, TLRs are primarily localised in intestinal epithelial cells (IECs) [42] and intestinal lamina propria macrophages [43, 44]. Most TLRs, such as the lipopolysaccharide (LPS)-receptor TLR4 [32, 43, 45, 46], detect their ligands at the cell surface. In the healthy intestine, TLR4 serves to keep the tolerance of the intestinal immune system to commensal bacteria [42], to maintain mucosal homeostasis [47], and to prevent allergic reaction to food antigens [48]. In active IBD, TLR4 expression is significantly increased in IEC as well as in lamina propria mononuclear cells (LPMNCs) [43, 45]. Several mutations within the TLR4 gene locus have been associated with IBD [32, 33] and an increased susceptibility to IBD has been identified for coexistent mutations within the TLR4 and the NOD2 gene [49]. Activation of TLR4 results in the activation of various signal transducers, such as nuclear factor kappa B (NF-*κ*B), signal transducer and activator of transcription 1 (STAT1), mitogen-activated protein kinases (MAPKs) or PPAR*γ*, with pro- as well as anti-inflammatory effects. As a functional consequence, TLR4 stimulates the expression of cytokines, such as TNF, IL1*β*, and IL6 via NF-*κ*B or STAT1 [50]. In contrast, increased TLR4-induced PPAR*γ* activity results in subsequent uncoupling of NF-*κ*B target genes as a part of a negative feedback mechanism and therefore limits inflammation [51, 52]. Studies in mice support the hypothesis that TLR4 mutations elevate the receptor function and promote intestinal inflammation via excessively activated cytokine-secretion [53, 54], possibly due to an increased activity of the receptor in response to physiological LPS concentrations. Additionally, mutations within the TLR4 gene locus can also lead to a functional loss of TLR4 that worsens DSS-induced colitis in mice by disturbing the intestinal homeostasis and barrier function [47, 53]. Thus, dysfunction of TLR4 in both directions aggravates intestinal inflammation. 

A recent study showed that TLR4 specifically activates the transcription factor X-box-binding protein-1 (XBP1), which is part of the unfolded protein response (UPR) cascade that is initiated in response to endoplasmatic reticulum (ER) stress of the cell [55]. The UPR consists of three signalling pathways, namely inositol-requiring enzyme 1 *α* and *β* (IRE1*α* and *β*) whose activation leads to increased XBP-1 function, protein kinase-like ER kinase (PERK), and activating transcription factor 6 (ATF6). The UPR then is responsible for folding, processing, export, and degradation of proteins during ER stress. An SNP within the XBP-1 gene has also been associated with IBD and loss of the protein is followed by Paneth-cell deficiency and increased levels of TNF*α* and flagellin in mice [56]. Further, XBP-1 is required for an appropriate response of TLR-4 to its ligands [55]. These observations indicate that ER stress may contribute to the pathogenesis of IBD, that is, by genetically caused XBP-1 dysfunction. On the other hand, ER stress seems also to be a common consequence of chronic inflammatory conditions in the intestine. This latter hypothesis is supported by observations showing that the ER stress response is induced in IL-10 deficient mice and in animals featuring an aberrant mucin assembly [57, 58].

## 5. NOD2—from Microbiota to Defence

NOD2 represents probably the best investigated and most well-established CD susceptibility gene [30, 31]. NOD2 consists of a C-terminal leucine-rich repeat domain (LRR), which is responsible for the antigen recognition, an intermediate nucleotide-binding domain (NBD) for oligomerization and signal transduction, and two protein-interaction domains, the caspase-activating and recruitment domains (CARDs). NOD2 is strongly expressed in colonic epithelial cells and Paneth cells in the small intestine as well as in intestinal macrophages in the small and large intestine. So far, only one ligand has been identified, namely muramyl-dipeptide (MDP) [59, 60], a wall component of gram-negative as well as gram-positive bacteria that is transported by the brush border transporter, human peptide transporter 1 (hPepT1), across the apical cell membrane [61]. NOD2 recognizes its ligand in the cytosol and, subsequently, directly interacts with its target molecules causing an activation of the innate immune system [62]. Activation of NOD2 in the uninflamed intestine results mainly in the induction of three different downstream effects. First, the activation of the transcription factor NF-*κ*B, followed by an increased expression of proinflammatory cytokines, such as TNF or IL1*β*. Secondly, the induction of caspase-mediated apoptosis [46], and thirdly, an increase in the expression level of antimicrobial peptides, such as the human defensins [63, 64]. 

Interestingly, NOD2 expression is increased in intestinal biopsies from CD patients. This may be caused by the proinflammatory cytokines TNF and interferon gamma (IFN*γ*) that are able to induce NOD2 expression in IEC [65]. This indicates that the innate immune system can increase its alertness for bacterial translocation or invasion. Mutations within the NOD2 gene have only been associated with CD, but not UC, and are present in about 40% of CD patients. Especially three specific mutations have been linked to an elevated susceptibility to CD [30, 31]. In particular, SNP8, SNP12 (both representing missense mutations), and SNP13 (a frame-shift mutation) are independently correlated with an early onset and an ileal localisation of the disease [66, 67]. The CD-associated mutations are located within the LRR domain of NOD2 and surprisingly cause a decreased activation of NF-*κ*B in response to MDP in vitro [59, 60]. 

**Figure 2:**
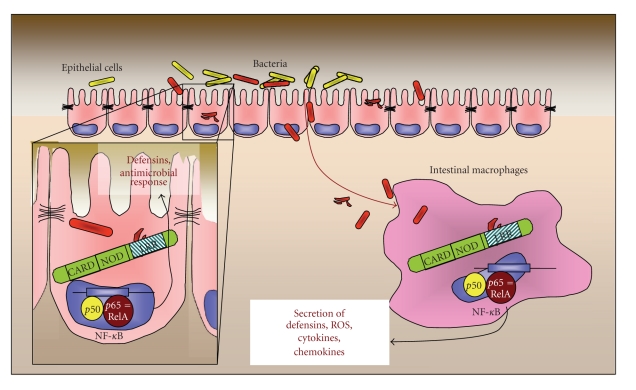
NOD2 and intestinal immune response. The NOD2 contains an effector-binding domain (CARD), a self-oligomerization domain (NOD), and a ligand recognition domain (LRR). The three CD-associated SNPs are located within or near the LRR domain. NOD2 is primarily localised in intestinal epithelial cells and macrophages. Upon binding to its ligand, bacterial MDP, NOD activates the transcription factor NF-*κ*B, what mainly results in the expression of the antimicrobial defensins and various cytokines that trigger the antimicrobial response.

These observations suggest that mutant NOD2 is not able to activate NF-*κ*B adequately, what might result in a pathological and insufficient immune response of the intestinal epithelium to microbial contact and stimulation. Interestingly, and contrary, intestinal lamina propria macrophages from CD patients feature a highly induced expression of NF-*κ*B-dependent, proinflammatory mediators, such as IFN*γ*, TNF, and IL1*β* [46]. These observations are corroborated by studies in MDP-stimulated macrophages from NOD2 mutant mice. The animals feature increased and constitutive NF-*κ*B activation, increased IL1*β* secretion, and elevated apoptosis rates [68]. Additionally, in biopsies from CD patients, far more intracellular and epithelial-adherent bacteria are detectable as compared to biopsies derived from healthy controls [69]. These findings provide a possible mechanism how a defective NOD2-variant could cause a dysregulated immune response in the intestinal epithelium and therefore essentially contribute to the onset of CD; Stimulation by MDP causes overwhelming activation of a defective NOD2 variant in intestinal macrophages. Subsequent overactivation of NF-*κ*B results in increased secretion [46] of proinflammatory mediators, such as TNF and IFN*γ* that lead to increased expression of NOD2 in IEC. However, due to the mutation, the epithelial isoform of NOD2 is not able to detect and respond to the bacterial stimuli adequately, what results in an inappropriate level of cytokine secretion, uncontrolled inflammatory conditions, a reduced amount of secreted antimicrobial peptides, such as human defensins, and, finally, in an insufficient response to intestinal bacteria (Figure 2).

## 6. The Microbiota and Graft versus Host Disease

Additionally, recent data also indicate a pivotal role of the IBD-associated NOD2 SNPs in the pathogenesis of graft versus host disease (GvHD), one of the most deleterious complications after allogenic, haematopoietic stem cell transplantation (HSCT). Holler et al. demonstrated an increased risk for the development of GvHD and an elevated treatment-related mortality when the donor or the host was carrying the IBD-associated mutations within the NOD 2 gene [70, 71]. A further study, analyzing the effects of each of the NOD2 SNPs separately, indicated that the clinical manifestation of GvHD might be critically dependent on the presence of SNP13 in the donor [72]. In contrast, it appeared in a different investigation that the risk for the onset of GvHD is reduced when only the donor is carrying the NOD2 variants [73]. A mechanistic explanation, how NOD2 mutations could contribute to increased risk for the development of GvHD and to elevated treatment-related mortality in HSCT patients, could be presented by the finding that NOD2 plays an important role for the regulation of host antigen-presenting cells (APCs). NOD2 dysfunction in host APC causes an increased proliferation and activation of donor T-cells finally resulting in the onset of GvHD, bacteraemia, and, at least in a mouse model, increased intestinal inflammation [74, 75].

## 7. Human Defensins—The Innate Antimicrobiant

As outlined above, the number of epithelial surface bacteria is increased in CD. These observations lead to the hypothesis that the antimicrobial defence mechanisms in the intestine of CD patients could be impaired. The small intestine, especially the ileum, also represents the home of the Paneth cells with their main innate antimicrobial effector molecules, the human defensins (HDs) 5 and 6. Therefore, it seems plausible that a diminished expression or function of the HD contributes to an impaired innate host defence to bacteria and to the onset of disease. So far, ten HDs have been identified that are separated into two groups, six *α*-defensins and four *β*-defensins, being the *α*-defensins HD5 and HD6 the most important in the intestinal mucosa [76]. On a functional level, the defensins exert bactericidal activity, since they are able to form micropores in the bacterial wall resulting in collapse and death of the bacterium [77]. In the small intestine, HD5 and HD6 are mainly expressed in the Paneth cells at the base of the crypts of Lieberkühn, whereas the *α*-defensins 1-4 are produced by lamina propria neutrophils [78, 79]. In the colon, the human *β*-defensins (HBD-1-4) are produced and secreted by IEC and lamina propria plasma cells [63]. The constitutively expressed HD5 and HD6 are believed to contribute essentially to the maintenance of intestinal epithelial barrier function by protecting the intestinal stem cells that are located in the vicinity of the Paneth cells [80]. Wehkamp et al. demonstrated a decreased expression of HD5 and HD6 in patients with ileal CD compared to control patients [81]. They further elucidated that CD patients featuring NOD2 mutations showed even lower levels of HD5 mRNA compared to CD patients with wild-type NOD2. These findings indicate that NOD2 mutants are closely related to altered (decreased) levels of the human defensins and subsequent impaired antimicrobial activity in the small intestine, since NOD2 is also highly expressed in Paneth cells, and NOD2 mutations are also associated with ileal disease. However, the defensin promoter region lacks a binding site for the main transcription factor that is activated by NOD2, NF-*κ*B [82]. 

## 8. The Inflammasome—A Sensor for Invasive Bacteria?

One of the biggest advantages of the innate immune system is its ability to respond rapidly and persistently to pathogenic conditions. Though the innate immune system is genetically programmed and reacts always similar to a stimulating agent, it plays a crucial role not only for early host defence but also for the activation of the adaptive immune system and for the induction of acquired immune responses. A key role herein plays the NOD-like receptor (NLR) family. Members of that protein family can form the so-called inflammasomes. These multiprotein complexes activate caspase-1 resulting in the expression and secretion of inflammatory mediators, such as IL1*β* or IL18. One of the best described members of the respective family is the Pyrin domain containing NLR3 (NALP3). The NALP3 inflammasome is composed of NALP3, caspase-1, and the adaptor molecule ASC [83]. The final assembly of the inflammasome leads to the self-activation of caspase-1 resulting in the activation of the proinflammatory cytokines IL-1*β* and IL-18 [84–86]. Among the activators of NALP3 are vaccine adjuvants, such as alum [87–90], bacteria, such as Listeria monocytogenes [91, 92] and MDP. In addition to the widely known NOD2-NF-*κ*B-mediated activation of IL1*β*, MDP is also able to induce the interleukin level via increasing the activity of caspase-1 and NALP3 in human monocytes, suggesting that NALP3 acts as an additional MDP sensor [93, 94]. Mouse studies revealed that activation and secretion of IL1*β* is dependent on the activity of both of the regulatory factors, NOD2 and NALP3 [95]. These findings are corroborated by studies using monocytes from CD patients showing that a dysfunction mutation within the NOD2 gene prevents MDP-induced upregulation of IL1*β* [96, 97], whereas a gain of function mutation within the NALP3 gene in Muckle-Wells patients as well as a respective mutation in mice causes overexpression of IL1*β* [68, 93]. In addition, in vitro studies showed that the NALP1 inflammasome is also sensitive to MDP and might be involved in MDP-induced IL1*β* expression [98, 99]. Though the knowledge of the exact role of the NALP inflammasomes in the immune system is just rudimentary so far and needs to be further elucidated, these findings suggest that the NALPs might be essentially involved in the pathogenesis of intestinal inflammation.

## 9. Autophagy Genes—More Than Just Cleaning the Cell?

Autophagy represents an essential intracellular process that is responsible for the turnover of protein aggregates, the removal of damaged organelles, and the elimination of intracellular microbes. Therefore, autophagy can also be regarded as a part of the innate immune system [100]. Recent GWAS showed a significant association of the autophagy gene, ATG16L1 and IRGM with CD [28, 29, 36, 101]. So far, only little is known about the role and function of the autophagy genes in the intestinal epithelium. The Thr300Ala substitution polymorphism within the ATG16L1 gene is associated with an ileal CD phenotype, similar as the CD-associated NOD2 mutations [65, 102] and a specific E. coli strain, adherent-invasive E. coli [103, 104], which are able to survive and to replicate within intestinal macrophages [105]. A recent study demonstrated that loss of either ATG16L1 or IRGM contributes to increased replication and survival of the specific E. coli strains in vitro [106], a finding that is in good accordance with the results of previous studies demonstrating prolonged survival of Salmonella typhimurium in human epithelial cells [107, 108]. Moreover, ATG16L1 seems to play a major role for the correct function of the intestinal Paneth cells that also represent one of the main intestinal localisations of NOD2 and the main source of the human defensins. Cadwell et al. have recently revealed abnormalities in the Paneth cell granule exocytosis pathway and in the gene transcription profile in ATG16L1-deficient mice as well as in tissue specimen derived from CD patients carrying the CD risk allele [109]. Surprisingly, the ATG16L1 mutation caused, among others, an increased expression of genes involved in PPAR*γ* signalling. Of special interest with respect to the pathophysiology of CD is the finding that the exocytosis pathway of mutant-carrying Paneth cells is disrupted. Since the Paneth cells secrete the important antimicrobial defensins, these findings could essentially contribute to an aberrant innate immune response to microbial stimuli in the gastrointestinal tract and therefore play a pivotal role for the onset of chronic intestinal inflammation. Recent studies also suggest that ATG16L1 is involved in the regulation of inflammasome activity [110] and interacts with NOD2 at the sites of bacterial cell invasion [111, 112].

## 10. PTPN2—A Likely Key Regulator of Intestinal Inflammation

A recently identified IBD-associated gene locus encodes for PTPN2 [29, 36]. By dephosphorylating and thereby inactivating its targets, the regulatory protein PTPN2 modulates and regulates proinflammatory signal transduction as induced by cytokines such as TNF, IFN*γ*, or IL6. Among its targets are the signal transducers and activators of transcription 1+3 (STAT1+3) [113–115], mitogen-activated protein kinases (MAPKs) [116], the epidermal growth factor receptor (EGFr) [117, 118], and the insulin receptor [119]. PTPN2 knock-out (PTPN2−/−) mice feature excessively high levels of TNF, IL12B, and IFN*γ*. The importance of PTPN2 for the regulation of inflammation in vivo is further corroborated by the observation that PTPN2−/− mice are not able to survive longer than 3 to 5 weeks, finally dying on a progressive systemic inflammatory syndrome [120, 121]. Of special interest with respect to CD is the fact that PTPN2−/− mice develop severe diarrhoea and weight loss, both of them representing common symptoms in human CD. Additionally, PTPN2−/− mice represent systemic hyperresponsiveness to TLR4 ligand, LPS, resulting in increased production of IFN*γ* and nitric oxygen (NO) [120, 122] that are also major pathogenetical factors in CD. These observations suggest that PTPN2 might play an important role for the adequate reaction of the innate immune system to bacterial stimuli. The possible importance of PTPN2 for human disease has been underlined by a recent study using IFN*γ*-treated IEC [123]. Here, it has been demonstrated that PTPN2 downregulates IFN*γ*-induced proinflammatory STAT1 signalling. From a functional perspective, loss of PTPN2 permitted IFN*γ* to increase the expression of the pore-forming protein, claudin-2, resulting in a dramatic decrease of the intestinal epithelial barrier function. These data, in addition to the previously identified role for PTPN2 in regulating immune signalling, provide the rationale background for a functional role of the regulatory protein PTPN2 in the pathogenesis of IBD, assumingly by regulating cytokine signalling and innate immune responses as well as in preserving the intestinal epithelial barrier function. 

## 11. Conclusions

**Figure 3:**
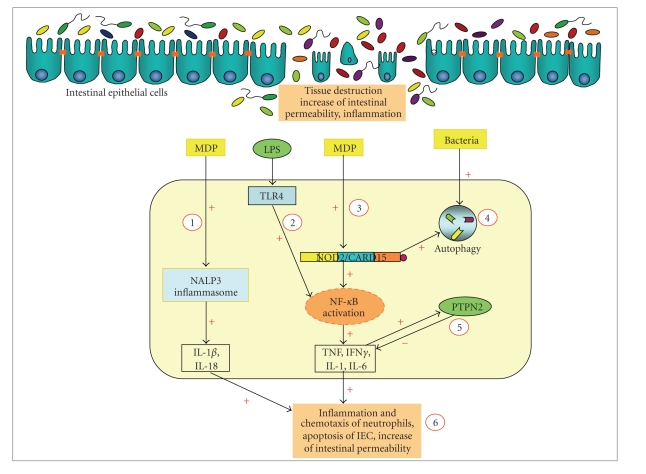
The innate immune system during intestinal inflammation. A defect in the intestinal epithelium, possibly genetically driven, causing tissue destruction, increased epithelial permeability and inflammation permits bacteria and their antigens, such as LPS and MDP, to penetrate through the epithelial monolayer. (1) The bacterial wall component, peptidoglycan, is cut by intracellular endosomes to MDP, that can activate the NALP3 inflammasome. As a consequence, pro-IL1*β* and pro-IL18 are processed to active molecules, what triggers proinflammatory conditions in the epithelium. (2) LPS binding to its receptor, TLR4, results in the activation of NF-*κ*B and, subsequently, in increased expression of cytokines, such as TNF, IFN*γ*, or IL6. (3) MDP activates NOD2 directly, causing increased NF-*κ*B activity. In addition to elevated cytokine levels, NOD2 also induces the expression of antimicrobial peptides, such as defensins. (4) Bacteria, such as *E. coli* or *Listeria monocytogenes*, can activate the autophagosome that plays a key role for inactivating invasive bacteria and other pathogenic components. The autophagy machinery is also regulated by NOD2 activity. (5) Cytokines, such as IFN*γ*, have been shown to increase the activity of PTPN2 that, in turn, downregulates proinflammatory signalling. Dysfunction of PTPN2 results in an impaired epithelial barrier function and elevated secretion of proinflammatory cytokines. (6) Malfunction of the innate immune response mechanisms in the gastrointestinal tract, possibly genetically triggered, causes tissue destruction, increased apoptosis of intestinal epithelial cells, elevated epithelial permeability, and, finally, establishes a chronic inflammatory state in the intestine.

The innate immune system plays a pivotal role for the control of the intestinal mcirobiota. On the other hand, the human microbiota regulates the innate immune system (Figure 3). Our increasing understanding of the molecular mechanisms that modulate the innate immune response to bacterial and antigen in the intestine are also raising about the complex signalling and networking. Further understanding of the pathways how the intestinal microbiota contributes to the pathophysiology of chronic intestinal inflammation will help us to develop new therapeutic strategies.
